# Supplementary material for: Health disparities, and health behaviours of older immigrants & native population in Norway
Source: PLoS One. 2022 Jan 31;17(1):e0263242. doi: 10.1371/journal.pone.0263242 (PMC8803195; doi:10.1371/journal.pone.0263242)
Supplement: Attachment — Submitted filename: Response_Reviewers.docx [file pone.0263242.s001.docx]

**Response Reviewers**

Journal Requirements:

When submitting your revision, we need you to address these additional requirements.

1. Please ensure that your manuscript meets PLOS ONE's style requirements, including those for file naming. The PLOS ONE style templates can be found at 
<https://journals.plos.org/plosone/s/file?id=wjVg/PLOSOne_formatting_sample_main_body.pdf> and 
<https://journals.plos.org/plosone/s/file?id=ba62/PLOSOne_formatting_sample_title_authors_affiliations.pdf>

**Response:** Done.

2. Please review your reference list to ensure that it is complete and correct. If you have cited papers that have been retracted, please include the rationale for doing so in the manuscript text, or remove these references and replace them with relevant current references. Any changes to the reference list should be mentioned in the rebuttal letter that accompanies your revised manuscript. If you need to cite a retracted article, indicate the article’s retracted status in the References list and also include a citation and full reference for the retraction notice.

**Response:** Done.

3. Please change "female” or "male" to "woman” or "man" as appropriate, when used as a noun (see for instance <https://apastyle.apa.org/style-grammar-guidelines/bias-free-language/gender>).

**Response:** Done

4. We note that you have indicated that data from this study are available upon request. PLOS only allows data to be available upon request if there are legal or ethical restrictions on sharing data publicly. For more information on unacceptable data access restrictions, please see <http://journals.plos.org/plosone/s/data-availability#loc-unacceptable-data-access-restrictions>.

In your revised cover letter, please address the following prompts:

a) If there are ethical or legal restrictions on sharing a de-identified data set, please explain them in detail (e.g., data contain potentially sensitive information, data are owned by a third-party organization, etc.) and who has imposed them (e.g., an ethics committee). Please also provide contact information for a data access committee, ethics committee, or other institutional body to which data requests may be sent.

b) If there are no restrictions, please upload the minimal anonymized data set necessary to replicate your study findings as either Supporting Information files or to a stable, public repository and provide us with the relevant URLs, DOIs, or accession numbers. For a list of acceptable repositories, please see <http://journals.plos.org/plosone/s/data-availability#loc-recommended-repositories>.

We will update your Data Availability statement on your behalf to reflect the information you provide.

**Response:** As we have indicated in our previously published article from the same survey data *“Qureshi SA, Straiton M, Gele AA. Associations of socio-demographic factors with adiposity among immigrants in Norway: a secondary data analysis. BMC Public Health. 2020 May 24;20(1):772. doi: 10.1186/s12889-020-08918-9. PMID: 32448125; PMCID: PMC7247236.*

**Availability of data and materials:** The data is available from NSD (Norwegian Centre for Research

Data) upon request  [nsd@nsd.no](mailto:nsd@nsd.no).

**Ethics approval and consent to participate**: The data is available from NSD (Norwegian Centre for Research Data) upon request. We signed a privacy contract before we started analysing the data. The survey was approved by the regional ethical committee and the participants gave written consent. The

participants were free to withdraw at any time and their data was deleted immediately with no consequences.

**Consent for publication**: As our manuscript does not include any individual data or sensitive personal information, therefore consent for publication is “Not Applicable” in this case.

5. We noticed you have some minor occurrence of overlapping text with the following previous publication(s), which needs to be addressed:

- <https://www.mdpi.com/1660-4601/17/20/7387/html> 
- <http://docplayer.net/37706939-Health-of-immigrants-in-european-countries.html>
- <https://onlinelibrary.wiley.com/doi/10.1111/j.1747-7379.2008.00150.x>

The text that needs to be addressed involves the Discussion.

**Response:** We have rectified the overlapping text.

***Abstract:*** Line 52-54.

***Discussion:*** **pg 9**, *line235-236*. **Pg 13**, *line 318-322. Pg 13, line 323-333.*

In your revision ensure you cite all your sources (including your own works), and quote or rephrase any duplicated text outside the methods section. Further consideration is dependent on these concerns being addressed.

[Note: HTML markup is below. Please do not edit.]

Reviewers' comments:

Reviewer's Responses to Questions

**Comments to the Author**
1. Is the manuscript technically sound, and do the data support the conclusions?

The manuscript must describe a technically sound piece of scientific research with data that supports the conclusions. Experiments must have been conducted rigorously, with appropriate controls, replication, and sample sizes. The conclusions must be drawn appropriately based on the data presented.

Reviewer #1: Yes

2. Has the statistical analysis been performed appropriately and rigorously?

Reviewer #1: Yes

3. Have the authors made all data underlying the findings in their manuscript fully available?

The [PLOS Data policy](http://www.plosone.org/static/policies.action#sharing) requires authors to make all data underlying the findings described in their manuscript fully available without restriction, with rare exception (please refer to the Data Availability Statement in the manuscript PDF file). The data should be provided as part of the manuscript or its supporting information, or deposited to a public repository. For example, in addition to summary statistics, the data points behind means, medians and variance measures should be available. If there are restrictions on publicly sharing data—e.g. participant privacy or use of data from a third party—those must be specified.

Reviewer #1: Yes

4. Is the manuscript presented in an intelligible fashion and written in standard English?

PLOS ONE does not copyedit accepted manuscripts, so the language in submitted articles must be clear, correct, and unambiguous. Any typographical or grammatical errors should be corrected at revision, so please note any specific errors here.

Reviewer #1: Yes

5. Review Comments to the Author

Please use the space provided to explain your answers to the questions above. You may also include additional comments for the author, including concerns about dual publication, research ethics, or publication ethics. (Please upload your review as an attachment if it exceeds 20,000 characters)

Reviewer #1: The study addresses a relevant and emerging issue/challenge in public health: health disparities between immigrants and native population in European countries, especially in old age that is associated with functional decline and physical challenges that, in turn, compromise the ability to perform necessary tasks required for daily living.

INTRODUCTION:
As the Authors stated in the introduction, there are some previous studies, although not many, dealing with the same topic.
I do suggest to the Authors to include in the introduction some comments about other previous references that, to my opinion, it is worth mentioning. For instance, IJERPH in 2019 published a special issue dealing with "Migration, Work and Health", which included both some original papers and a scoping review addressing the topic of immigrant workers' health disparities in European countries.

**Response:** We appreciate the response of the reviewer and have now added additional comments in the introduction ***pg.3 line 68-73*** *“The number of immigrants in Europe, born outside the EU-states increased from* ***2,48 million in 2010 to 3,22 million in 2018 (5). There are around*** *95.7 million men and 68.1 million women migrant workers globally, these workers are a large part of the labor force particularly in Europe (6). Due to several challenges such as language and culture, migrants usually perform hazardous low paid jobs which the natives do not take up (7-9). These in turn lead to adverse health outcomes later in life which usually start at younger ages (6, 7, 10)”.*

And on ***pg 3 line 83-85*** *“According to a report from the Norwegian Institute of health (NIPH), today the ratio is 2:1 i.e two people working (16-64 years) for every person under 16 or over 64 years (14). This will drop to 1.6 by 2060 and further to below 1.4 by the turn of the century (14)”.*

DISCUSSION:
I do suggest to the Authors to add some comments about gender differences (if any) and some more details (e.g. some examples taken from the included surveys) about the negative effect of "3-D" jobs on the health of immigrants.

**Response: Again we agree with the reviewers comments and have now added additional text in the discussion section on pg. 11, lines 264-274**

***“****There is a clear difference in the socio-economic status in our survey data (34), according to which only 25% of immigrants work as managers, academicians (College, University), whereas the corresponding percentage in the general population is 54%. Immigrants are overrepresented in professions that do not require high educational levels such as cleaners, transport industry, auxiliary workers, construction industry etc (16, 34). Almost 53% of immigrant men have to work under risky conditions (noise, ergonomically unsafe, exposure to chemicals etc) as compared to 38% in the general population (16). This has also been reported by studies from other parts of the world, that immigrants have to work in unfriendly and risky environments leading to health hazards (6, 9, 35). However, there is a huge gender difference regarding the type of profession, as women are less involved in physical labor and more in jobs such as social care & health care workers (16, 34)”.*

6. PLOS authors have the option to publish the peer review history of their article ([what does this mean?](https://journals.plos.org/plosone/s/editorial-and-peer-review-process#loc-peer-review-history)). If published, this will include your full peer review and any attached files.


If you choose “no”, your identity will remain anonymous but your review may still be made public.


**Do you want your identity to be public for this peer review?** For information about this choice, including consent withdrawal, please see our [Privacy Policy](https://www.plos.org/privacy-policy).

Reviewer #1: No

[NOTE: If reviewer comments were submitted as an attachment file, they will be attached to this email and accessible via the submission site. Please log into your account, locate the manuscript record, and check for the action link "View Attachments". If this link does not appear, there are no attachment files.]

While revising your submission, please upload your figure files to the Preflight Analysis and Conversion Engine (PACE) digital diagnostic tool, <https://pacev2.apexcovantage.com/>. PACE helps ensure that figures meet PLOS requirements. To use PACE, you must first register as a user. Registration is free. Then, login and navigate to the UPLOAD tab, where you will find detailed instructions on how to use the tool. If you encounter any issues or have any questions when using PACE, please email PLOS at [figures@plos.org](mailto:figures@plos.org). Please note that Supporting Information files do not need this step.
